# Low-Pressure Chemical Vapor Deposition SiN_x_ Process Study and Its Impact on Interface Characteristics of AlGaN/GaN MISHEMTs

**DOI:** 10.3390/mi16040442

**Published:** 2025-04-09

**Authors:** Hu Sun, Qian Fan, Xianfeng Ni, Qiang Luo, Xing Gu

**Affiliations:** Institute of Next Generation Semiconductor Materials, Southeast University, Suzhou 215123, China; 220224561@seu.edu.cn (H.S.); 103200035@seu.edu.cn (Q.F.); 103200036@seu.edu.cn (X.N.); q.luo@seu.edu.cn (Q.L.)

**Keywords:** LPCVD SiN_x_, 2DEG density, gas flow ratio, gate dielectric, interface trap density, passivation layer

## Abstract

This study employed low-pressure chemical vapor deposition (LPCVD) SiN_x_ as both the gate dielectric layer and surface passivation layer, systematically investigating the effects of different growth conditions on the dielectric layer quality, two-dimensional electron gas (2DEG) characteristics, interface trap density, and devices’ performance, thereby optimizing the growth parameters of LPCVD SiN_x_. The experiment investigated the effects of growth parameters such as the growth temperature, chamber pressure, and gas flow ratio on the growth rate of SiN_x_ during the process of growing SiN_x_ using the LPCVD technique. Further studies were performed to analyze the impact of SiN_x_ introduction on the 2DEG performance. The results indicated that both Si-rich and N-rich SiN_x_ compositions could enhance the 2DEG density improvement induced by SiN_x_ passivation. The impact of the gas flow ratio on the interface trap density is studied. Through the quantitative characterization of the interface trap density using the pulse-mode I_DS_-V_GS_ method and frequency-dependent capacitance–voltage (C-V) measurement, the results show that the interface trap density decreases with an increased Si-to-N ratio.

## 1. Introduction

Gallium nitride (GaN) has emerged as a highly sought-after material in the research domain of semiconductor devices, owing to its remarkable characteristics such as a high electron saturation velocity, wide bandgap, high critical breakdown field strength, and high operating temperature [1]. AlGaN/GaN high-electron-mobility transistors (HEMTs) based on GaN materials have demonstrated dominant performance in high-frequency and high-voltage applications [2]. However, during HEMT operation, the “virtual gate” effect formed at the AlGaN surface can deplete portions of the channel two-dimensional electron gas (2DEG), leading to reduced device output current and current collapse. To mitigate the adverse effects of the “virtual gate” phenomenon while suppressing the gate leakage current and enhancing gate swing, depositing a material layer on the AlGaN surface as both a passivation layer and gate dielectric has been widely studied [3].

At present, the surface passivation of AlGaN has been extensively studied. A series of materials have been used as passivation layers and dielectric layers for GaN MISHEMT devices, including but not limited to Al_2_O_3_, SiO_2_, HfO_2_, SiN_x_, etc. [4,5,6,7]. Among a wide variety of passivation materials, SiN_x_, due to its unique properties, has become the most commonly used passivation material for GaN HEMTs in industry. SiN_x_ has been prepared by several techniques, including plasma-enhanced chemical vapor deposition (PECVD) [8], metal–organic chemical vapor deposition (MOCVD) [9,10], and low-pressure chemical vapor deposition (LPCVD) [11,12]. Each of these techniques has its own unique process parameters and applicable scenarios, providing diverse options for the effective application of SiN_x_ in GaN MISHEMT devices. In recent years, LPCVD SiN_x_ has been widely used in AlGaN/GaN MISHEMTs as gate dielectric and surface passivation layers. Hua et al. used LPCVD to deposit SiN_x_ films at 780 °C as the gate dielectrics of AlGaN/GaN MISHEMTs [13]. Liu et al. deposited 20 nm LPCVD SiN_x_ at 650 °C as the gate dielectric, achieving high on–off current ratios and high breakdown voltages [14]. Although SiN_x_ prepared by LPCVD has been proven to be suitable for use as gate dielectric and passivation layers in AlGaN/GaN MISHEMTs, it has also been reported that the insertion of such a gate dielectric layer leads to a high density of traps at the interface between the SiN_x_ dielectric layer and the III-nitride. The charging and discharging behavior of these interface traps during device operation can have an adverse impact on the device’s performance and stability [15].

In this paper, we focus on LPCVD SiN_x_, comparing the effects of different growth parameters on the SiN_x_ growth rate and investigating the impacts of SiN_x_ films with varying qualities on 2DEG characteristics. Based on the influence of SiN_x_ grown under different gas flow ratios on the 2DEG density, a detailed analysis is conducted to explore the underlying mechanisms responsible for the SiN_x_-induced increase in the 2DEG density. The pulse-mode I_DS_-V_GS_ method and the frequency-dependent C-V method were employed to characterize the interface traps quantitatively. Its impact on AlGaN/GaN MISHEMT devices’ performance is also reported.

## 2. Materials and Methods

**Figure 1:**
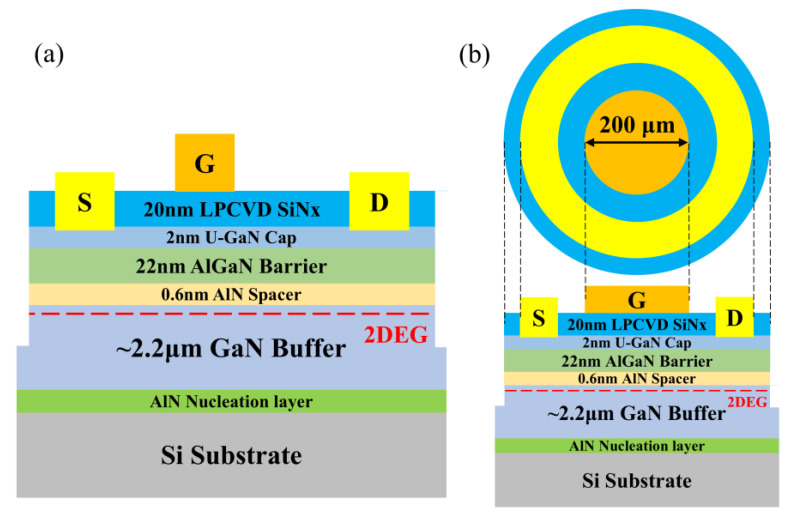
Schematic diagrams of the LPCVD SiNx/AlGaN/GaN (**a**) MISHEMT and (**b**) MIS diode.

The AlGaN/GaN HEMT structure was grown on a 6-inch Si (111) substrate via MOCVD by Suzhou Hanhua Ltd., Suzhou, China. The epitaxial structure, from bottom to top, consisted of an AlN nucleation layer, a 1.8 μm high-resistivity AlGaN/GaN buffer layer, a 350 nm GaN channel layer, a 0.6 nm AlN insertion layer, a 22 nm AlGaN barrier layer, and a 2 nm GaN cap layer. Finally, the grown wafer was divided into multiple samples, which were then cleaned and placed in an LPCVD system to deposit SiN_x_ under different growth conditions. Inductively Coupled Plasma Etching (ICP) technology was employed to perform dry etching on the unprotected SiN_x_ and III-nitride to achieve mesa isolation. After the mesa isolation process was completed, dry etching was utilized again to precisely open the ohmic contact areas defined by the second photolithography mask. Subsequently, an ohmic metal layer composed of Ti/Al/Ni/Au with thicknesses of 20 nm, 130 nm, 50 nm, and 50 nm, respectively, was deposited by electron beam evaporation. Thereafter, the samples were placed in a N_2_ atmosphere at 850 °C for a 30 s rapid annealing treatment. The contact resistance of the ohmic contact was accurately measured using the Transmission Line Model (TLM) method, and the measured value was 0.55 Ω·cm^2^. Through comparative analysis, this measured value was found to be at a comparable level to the data reported in the previous literature [16,17]. In the final stage of device fabrication, the gate region was patterned using the third photolithography mask, and then a gate metal layer of Ni/Al with thicknesses of 50 nm and 500 nm, respectively, was deposited by electron beam evaporation. For the fabricated device, the gate length was set to 2 μm, the gate width was 100 μm, the distance between the gate and the source was 2 μm, and the distance between the gate and the drain was 11 μm. Figure 1 depicts the schematic diagrams of the fabricated LPCVD SiN_x_/AlGaN/GaN MISHEMT and MIS diode.

To characterize the quality of the LPCVD SiN_x_, the thickness and refractive index of SiN_x_ were determined using a spectroscopic ellipsometer. The microtopography of the samples was observed, and the surface roughness after SiN_x_ growth was measured by atomic force microscopy (AFM) with a scanning range of 5 μm × 5 μm. The 2DEG properties including the 2DEG density, mobility, and sheet resistance were measured at room temperature via the Van der Pauw Hall measurement method using a Hall effect tester (HL9900, Toho, Nagoya, Japan). Additionally, the Keithley 4200 semiconductor parameter analyzer (Keithley 4200A-SCS, Tektronix, Beaverton, OR, USA) was employed to evaluate the electrical performance of the MISHEMTs and characterize the interface state density at the gate dielectric/III-nitride interface.

## 3. Results

### 3.1. Modulation of LPCVD SiN_x_ Growth Conditions

**Figure 2:**
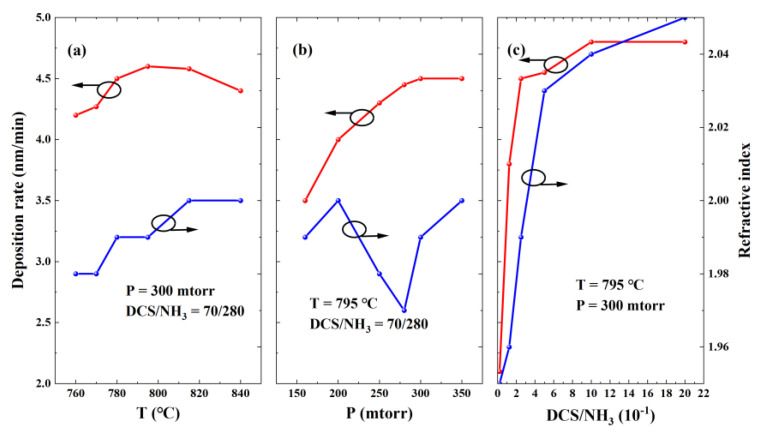
Dependences of deposition rate (red line) and refractive index (blue line) on (**a**) temperature, (**b**) pressure, and (**c**) DCS/NH_3_ ratio.

The quality of LPCVD SiN_x_ films is primarily governed by three growth parameters: the growth temperature (T), chamber pressure (P), and reactant gas flow ratio (SiH_2_Cl_2_ (DCS)/NH₃). The relationship between the deposition rate and these parameters was systematically investigated. Figure 2a illustrates the SiN_x_ deposition rate as a function of the growth temperature. Over the temperature range from 760 °C to 795 °C, the deposition rate increased monotonically due to the enhanced decomposition efficiency and reactivity of the DCS and NH_3_ precursors, which accelerated chemical reactions on the AlGaN surface. However, further temperature elevation (795 °C to 840 °C) resulted in a decline in the deposition rate, most likely attributed to the premature gas-phase decomposition of precursors that reduced the availability of reactive species for film formation [18]. Figure 2b demonstrates a direct proportionality between the SiN_x_ deposition rate and the gas flow ratio. As shown in Figure 2c, a similar positive correlation exists between the deposition rate and the chamber pressure, consistent with prior research findings [18,19].

The refractive index is a critical metric for evaluating the quality of LPCVD SiN_x_ films. To investigate the relationship between the quality of SiN_x_ and the growth parameters, the refractive index of the aforementioned samples was measured. Figure 2 illustrates the dependence of the SiN_x_ refractive index on the temperature, pressure, and gas flow ratio, with the gas flow ratio exhibiting the most significant influence. To further investigate the influence of the SiN_x_ growth parameters on the characteristics of the 2DEG, seven samples with SiN_x_ layers of different qualities grown by LPCVD were selected for subsequent research. Meanwhile, a sample without SiN_x_ was chosen as a reference sample. All these seven samples were taken from the same epitaxial wafer. The specific growth parameters are summarized in Table 1.

### 3.2. Influence of LPCVD SiN_x_ Growth Conditions on 2DEG

The 2DEG density (N_s_), sheet resistance (R_s_), and electron mobility (µ_n_) results of Samples A, B, C, D, E, F, and G are presented in Table 1. Compared with the sample without a SiN_x_ dielectric layer, a significant enhancement in the 2DEG density was observed in samples with deposited SiN_x_. Some viewpoints hold that the introduction of the passivation layer leads to an enhancement in the piezoelectric polarization effect caused by the additional strain in the AlGaN barrier layer and the entry of ionized electrons from Si donors into the channel, which results in an increase in the 2DEG density [20,21]. It has been reported that the diffusion of Si can only be achieved under high-temperature conditions [21,22]. To validate the above hypothesis, three epitaxial wafers were used as samples to investigate the changes in the 2DEG density before and after SiN_x_ growth via PECVD. The SiN_x_ growth conditions were consistent for all three samples. The N_s_, R_s_, and µ_n_ results are shown in Table 2. Consistent with the LPCVD SiN_x_ outcomes, samples with PECVD SiN_x_ exhibited significant 2DEG density increases. However, the high-energy ions in the PECVD process damage the surface of AlGaN/GaN, leading to an increase in defects. These defects increase the scattering of carriers, which in turn results in a decrease in electron mobility [23]. The experimental results show that depositing SiN_x_ under low-temperature conditions can also increase the 2DEG density, indicating that the entry of ionized electrons from Si donors into the channel is not the main reason for the increase in the 2DEG density. By observing the 2DEG density results of Samples A, B, C, and D, it can be found that as the gas flow ratio increases, the 2DEG density in the AlGaN/GaN heterojunction channel gradually increases. However, Sample E does not seem to follow this rule. Its N_s_ reaches 0.84 × 10^13^ cm^−2^, which is higher than that of Sample A. Strain relaxation typically leads to the formation of defects such as dislocations and reduces the interface quality. Nevertheless, some studies have pointed out that appropriate strain relaxation may release local stress and, in turn, increase N_s_ [10,24].

Two different conclusions prove that the strain variation is not the main cause of the increase in the 2DEG concentration. Siddique et al. found that N-rich SiN_x_ has a lower dielectric constant (ε), which leads to a decrease in the surface potential at the interface. The decrease in the surface potential makes the ionization energy closer to the Fermi level (E_F_), thereby promoting the ionization of surface states and increasing the 2DEG density [25]. Meanwhile, for Si-rich SiN_x_, the reduction in the proportion of NH_3_ introduces more un-nitrided Ga dangling bonds, forming near-conduction-band states. This results in a decrease in the surface potential and an enhanced accumulation of 2DEG concentration [26]. Therefore, the decrease in the surface potential is the main cause of the increase in the 2DEG concentration. Compared with Sample B, the growth temperature of Sample F was reduced by 15 °C, resulting in moderate reductions in both N_s_ and µ_n_, while R_s_ correspondingly increased. Considering the effects of the two temperatures on the growth rate, this study concludes that SiN_x_ deposited under the 795 °C condition exhibits superior quality compared to the 780 °C deposition condition. Upon comparing the results of Samples B and G, it was found that variations in pressure had little influence on N_s_.

Table 1 demonstrates that the introduction of LPCVD SiN_x_ also exerted a significant influence on both µ_n_ and R_s_. After SiN_x_ deposition, notable enhancements were observed in µ_n_, accompanied by corresponding improvements in R_s_. The analysis of Samples A–D reveals that increasing the gas flow ratio during LPCVD SiN_x_ growth led to a gradual rise in µ_n_ from 1822 cm^2^/V∙s to 1992 cm^2^/V∙s, while R_s_ decreased from 496 Ω/□ to 373 Ω/□. These results demonstrate that optimizing the gas flow ratio in the LPCVD SiN_x_ process can effectively enhance the transport properties of the 2DEG channel.

**Figure 3:**
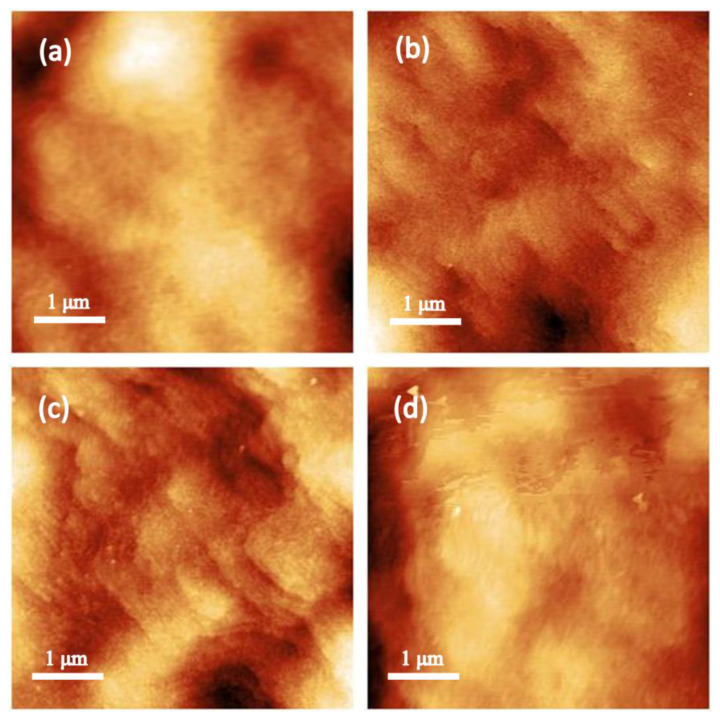
AFM images of AlGaN/GaN MISHEMTs for (**a**) Sample A, (**b**) Sample B, and (**c**) Sample C and (**d**) without SiN_x_.

The experimental results demonstrate that the gas flow ratio during LPCVD SiN_x_ growth exerts a significant impact on both the SiN_x_ film quality and 2DEG characteristics in the channel. To further investigate the influence of the gas flow ratio on the interface trap density and device electrical performance, Samples A, B, and C were selected for subsequent device studies. The SiN_x_ films prepared under three different gas flow ratios are in N-rich, standard Si/N ratio, and Si-rich states, respectively. Figure 3 shows the 5 μm × 5 μm AFM images of the AlGaN/GaN HEMT surface. Figure 3d is the AFM image of the unpassivated surface, with an AFM root mean square (RMS) roughness of 1.84 nm. Figure 3a–c are the AFM images of the passivated surfaces. The gas flow ratios (DCS/NH_3_) of Samples A-C are 1:8, 1:4, and 1:1, respectively, and their RMS roughness values are 1.36 nm, 1.55 nm, and 1.56 nm, respectively.

### 3.3. DC Characteristics of Devices

**Figure 4:**
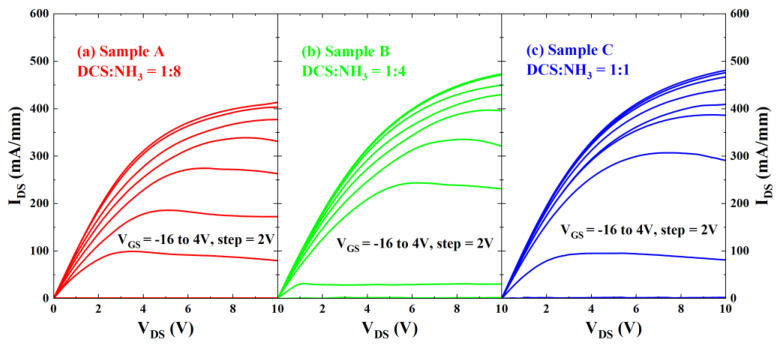
Output characteristic curves of AlGaN/GaN MISHEMTs for (**a**) Sample A, (**b**) Sample B, and (**c**) Sample C.

**Figure 5:**
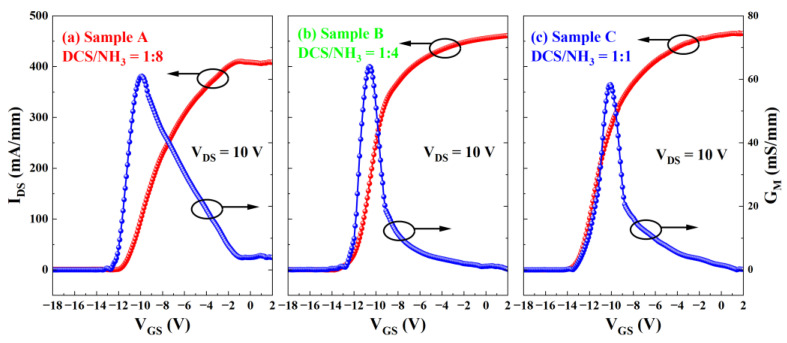
Transfer characteristic curves of AlGaN/GaN MISHEMTs for (**a**) Sample A, (**b**) Sample B, and (**c**) Sample C.

Figure 4 and Figure 5 present the output and transfer characteristics of the AlGaN/GaN MISHEMTs under DC conditions. The dimensions of the devices involved are W_G_/L_G_/L_GS_/L_GD_ = 100/2/2/11 μm. In the test depicted in Figure 4, the output curves of the devices are plotted from bottom to top, corresponding to gate voltages ranging from −16 V to 4 V with a step of 2 V. Figure 5 depicts the transfer characteristics of the three samples. In this measurement, the drain–source voltage V_DS_ was set at 10 V, and the gate–source voltage V_GS_ was scanned from −18 V to 2 V. The threshold voltages of Sample A, Sample B, and Sample C are −12.1 V, −13.2 V, and −13.4 V, respectively. It should be noted that while the set target thickness of SiN_x_ is identical, the samples are processed at different growth runs, which might cause some thickness difference which may be responsible for the Vth fluctuation mentioned above. It is believed that as the thickness of SiN_x_ increases, the threshold voltage exhibits a negative shift. Notably, in accordance with the research findings of Zhu et al. [26], the minor variations in the SiN_x_ thickness across different samples do not exert a significant influence on the interface trap density of AlGaN/GaN MISHEMTs. Moreover, there are no discernible differences in the peak transconductance (G_M_), which remains approximately 60 mS/mm for all samples.

**Figure 6:**
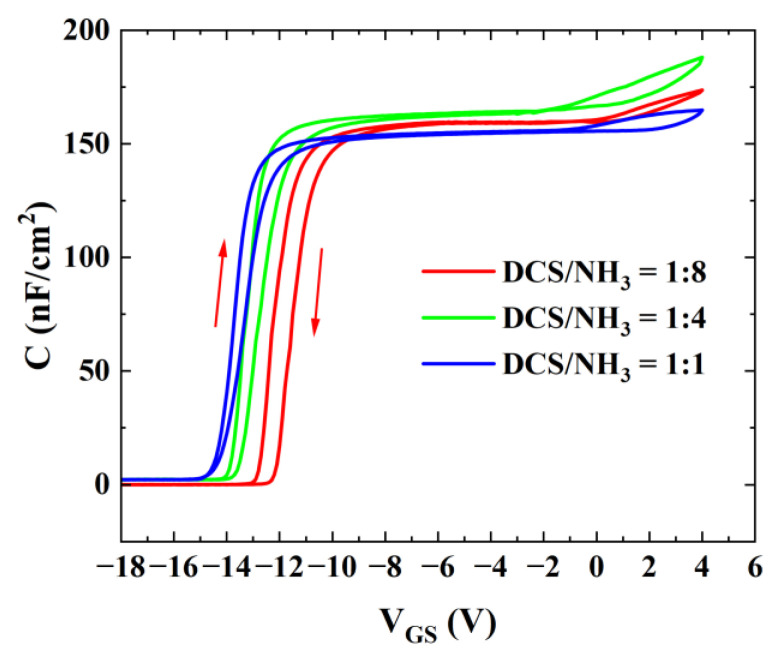
Capacitance measurements of AlGaN/GaN MIS diodes with three different gas flow ratios.

Figure 6 presents the capacitance–voltage (C-V) characteristic curves of AlGaN/GaN MIS diodes with three different gas flow ratios under normal temperature conditions. The gate diameter of these diodes was uniformly 200 μm. During the testing process, the gate voltage was first scanned forward from −18 V to 4 V in specific steps and then scanned backward from 4 V to −18 V at a 500 kHz frequency. Through an analysis of the experimental data and curves, it is clear that all three groups of samples exhibit the threshold hysteresis phenomenon, with varying degrees of hysteresis. This hysteresis is primarily attributed to the presence of numerous acceptor-like traps at the interface between the gate dielectric and III-nitride materials [15]. Sample A exhibited the largest hysteresis, while Sample C demonstrated the smallest, indicating that high-refractive-index SiN_x_ effectively reduces traps with long emission time constants at the interface.

### 3.4. Analysis of Gate Leakage Current

**Figure 7:**
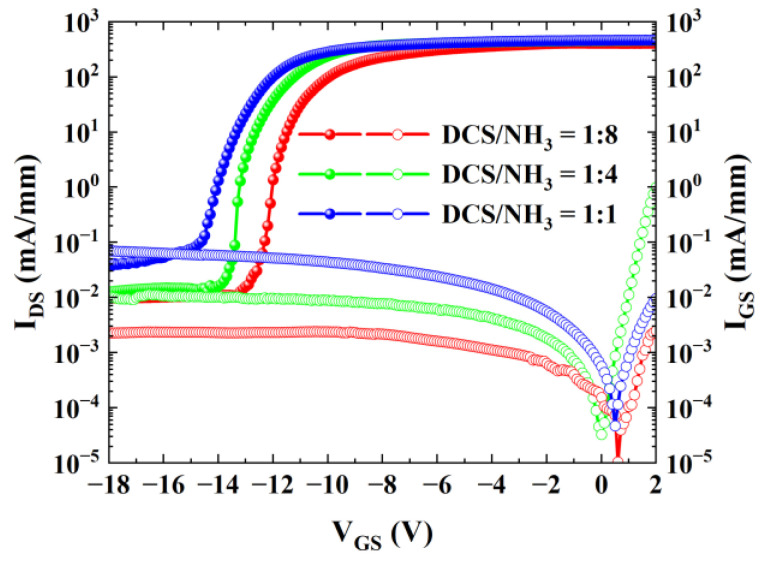
Transfer characteristic curves of AlGaN/GaN MISHEMTs for Sample A, Sample B, and Sample C in logarithmic coordinates.

As one of the crucial parameters of GaN HEMT devices, the gate leakage current exerts a vital influence on the device’s performance and reliability. Figure 7 depicts the curves of the drain current and gate current of the three samples as a function of the device gate voltage, with V_DS_ set at 10 V. It can be observed that as the gas flow ratio (DCS/NH_3_) gradually increases, the gate leakage current of the device exhibits an upward trend. This is primarily attributed to the fact that the increase in the Si source leads to an elevation in the concentration of Si donors at the interface between the dielectric layer and the III-nitride, thereby exacerbating the gate leakage phenomenon [21].

### 3.5. Interface Trap Characterization

Figure 6 indicates the existence of a large number of traps at the interface between the dielectric layer and III-nitride. To accurately evaluate the interface traps in AlGaN/GaN MISHEMTs and AlGaN/GaN MIS diodes, the pulse-mode I_DS_-V_GS_ method and the frequency-dependent C-V measurement were employed to characterize and analyze the distribution of interface states.

#### 3.5.1. Pulse-Mode I_DS_-V_GS_ Method

**Figure 8:**
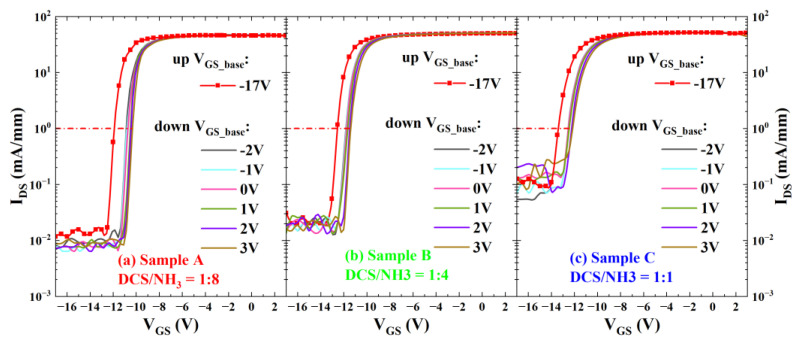
Pulse-mode I_DS_-V_GS_ characteristic curves of AlGaN/GaN MISHEMTs for (**a**) Sample A, (**b**) Sample B, and (**c**) Sample C.

The pulse-mode I_DS_-V_GS_ method is a common technique for characterizing interface traps in AlGaN/GaN MISHEMTs. In this study, to minimize the impact of the drain bias on the charge–discharge process of interface traps, the drain–source bias V_DS_ was set to 1 V. Figure 8 presents the pulsed-mode double-sweep curves of MISHEMT devices fabricated with three different gas flow ratios. Under the condition of V_DS_ = 1 V, when applying a forward scanning signal to the gate, the quasi-static gate bias voltage V_GS_base_ during forward scanning should be less than the device threshold voltage, thus set to −17 V. The forward sweep utilized a 500 ms period with a 50 μs pulse width and a maximum gate bias of V_GS_max_ = 3 V. For reverse sweeps, the quasi-static bias was typically chosen near the gate voltage where the output current approaches saturation to better characterize interface traps. As shown in Figure 8, the reverse-sweep V_GS_base_ was varied from −2 V to 3 V with a step of 1 V. Based on the difference in the threshold voltages between the forward and reverse scans of devices, the density of interface traps with an electron emission time constant greater than the pulse width can be determined. The density of interface traps D_it_ can be determined by the following formula:

(1) $$D_{\mathrm{it}}=\frac{C_{\mathrm{OX}\;}\cdot {\;\Delta V}_{\mathrm{TH}}}{q},$$

where the C_OX_ values of the three samples are 205 nF/cm^2^ (Sample A), 215 nF/cm^2^ (Sample B), and 200 nF/cm^2^ (Sample C), and ∆V_TH_ represents the difference in the threshold voltages between the forward and reverse scans.

The energy level depth (∆E_T_ = E_C_ − E_T_) corresponding to the electron emission time constant greater than the pulse width can be obtained from the Shockley–Read–Hall statistics [27]:

(2) $${\Delta E}_{T}=\mathrm{kTln}(v_{\mathrm{th}}\sigma_{n}N_{C}\tau_{e}),$$

where k is the Boltzmann constant, T is the absolute temperature, vth is the electron thermal velocity, $\sigma_{n}$ is the electron capture cross-section, and N_C_ is the effective density of states in the GaN conduction band. According to the calculation, under the condition that $\tau_{e}$ is 50 μs, ∆E_T_ = 0.438 eV. Therefore, the detectable energy range in this experiment is E_C_ − E_T_ ≥ 0.438 eV.

**Figure 9:**
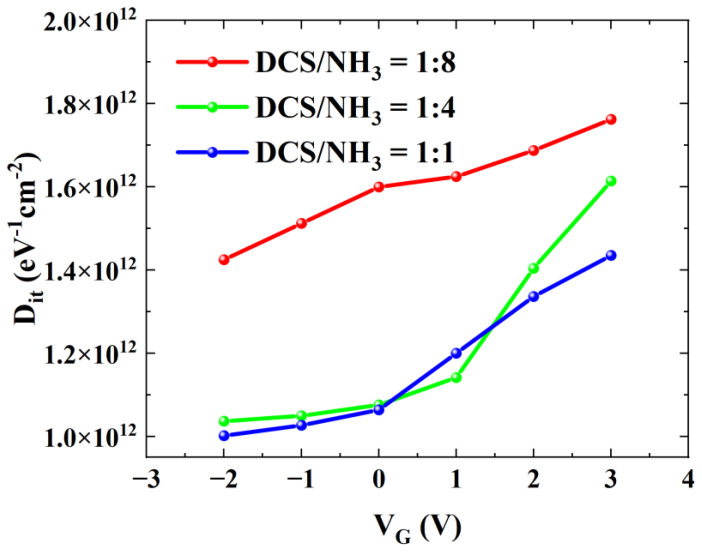
Interface state densities of AlGaN/GaN MISHEMTs with three different gas flow ratios obtained by pulse-mode I_DS_-V_GS_ method.

Figure 9 illustrates the interface trap density D_it_ extracted from the three samples under different reverse-scan V_GS_base_. When the reverse-scan V_GS_base_ is set to 3 V, the values of Sample A, Sample B, and Sample C are 1.76 × 10^12^ eV^−1^·cm^−2^, 1.61 × 10^12^ eV^−1^·cm^−2^, and 1.44 × 10^12^ eV^−1^·cm^−2^, respectively. The results indicate that as the value of the DCS/NH₃ ratio increases, the interface state density of the MISHEMT device gradually decreases. This is consistent with the results of the C-V measurements of the AlGaN/GaN MIS diodes presented previously.

#### 3.5.2. Frequency-Dependent C-V Measurement

While the pulse-mode I_DS_-V_GS_ method can characterize the interface state density in AlGaN/GaN MISHEMT devices, the presence of drain bias induces the partial detrapping of electrons from interface traps, leading to underestimated interface state density measurements. To achieve a more accurate characterization, frequency-dependent capacitance–voltage (C-V) measurement was employed in this study to further quantify the interface states at the dielectric/III-nitride interface.

**Figure 10:**
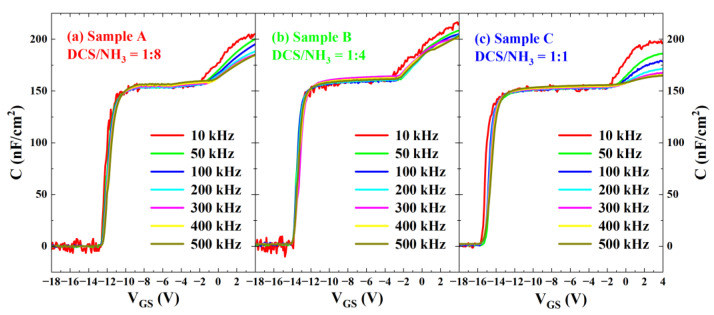
C-V curves of (**a**) Sample A, (**b**) Sample B, and (**c**) Sample C at different test frequencies.

Figure 10 shows the C-V curves of MIS diodes under three different gas flow ratios. The test frequencies f_m_ are set to 10 kHz, 50 kHz, 100 kHz, 200 kHz, 300 kHz, 400 kHz, and 500 kHz. In the test results, Sample A, Sample B, and Sample C all exhibit the characteristics of three steps and two ramps.

The Shockley–Read–Hall statistics (Equation (2)) describe the relationship between the electron emission time constant ($\tau_{e}$) and the corresponding energy level depth (E_C_ − E_T_). Given that the frequency-dependent C-V measurement characterizes the interface traps with frequency as a variable, we define the frequency corresponding to the interface trap emission time constant as the characteristic frequency f_it_.

(3) $$f_{\mathrm{it}}=\frac{1}{2\pi \tau_{e}}=\frac{v_{\mathrm{th}}\sigma_{n}N_{C}}{2\pi }\exp\left(-\frac{E_{C}\;-E_{T}}{\mathrm{kT}}\right)$$

With the continuous variation in the gate voltage, the degree of semiconductor energy-band bending exhibits corresponding dynamic changes. During this process, for the interface traps capable of responding to the alternating-current (AC) test signal (with the test frequency set as f_m_), their energy level depths will vary depending on the degree of energy-band bending. When a specific interface trap with an energy of E_T_ begins to respond to the test signal with a frequency of f_m_, the gate voltage precisely reaches the turn-on voltage V_ON_ of ramp 2. Under this condition, the following relationship necessarily holds:

(4) $$f_{\mathrm{it}}\left(E_{T}={\;E}_{\mathrm{Fs}}\right)=f_{m}$$

Yang et al. [28] accurately calculated the interface state density using the frequency-dependent C-V method. The interface state density can be obtained from the following equation:

(5) $$D_{\mathrm{it}}\left(E_{C}\;-\;E_{T}\right)=\frac{C_{\mathrm{ox}}\cdot {\Delta V}_{\mathrm{ON}}}{q\cdot {\Delta E}_{\mathrm{dis}}}-\;\frac{C_{\mathrm{ox}}+C_{B}}{q^{2}},$$

where C_OX_ is the capacitance of the dielectric layer, C_B_ is the capacitance of the barrier layer, ∆E_dis_ = ∆E_T_(f_1_,T_1_) − ∆E_T_(f_2_,T_2_), and ∆V_ON_ is the difference in the turn-on voltages of the C-V curves at two different frequencies.

**Figure 11:**
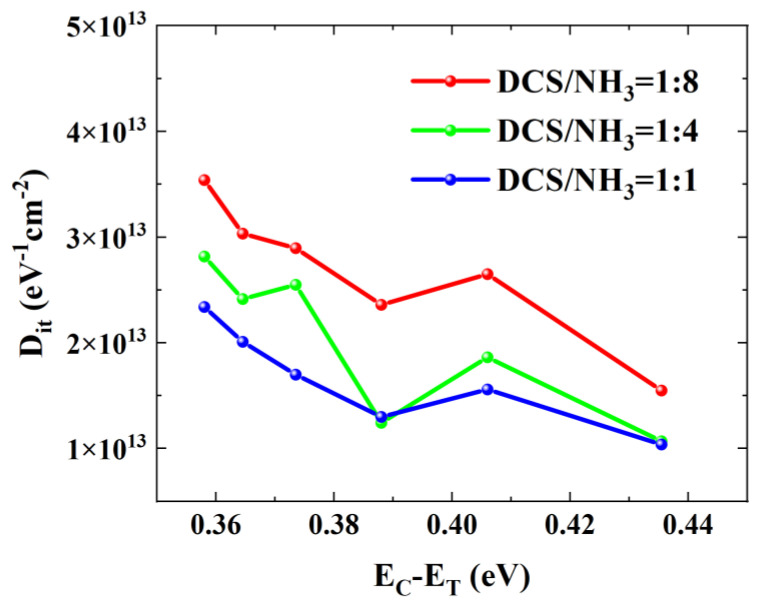
Interface state densities of AlGaN/GaN MISHEMTs with three different gas flow ratios obtained by frequency-dependent C-V measurement.

Figure 11 depicts the variation curves of the interface state density with the defect energy level depth for three samples. As can be seen from the figure, the interface state density shows a decreasing trend as the defect energy level depth increases. Specifically, for Sample A, the interface state density decreases from 3.54 × 10^13^ eV^−1^ cm^−2^ at a defect energy level of 0.358 eV to 1.55 × 10^13^ eV^−1^ cm^−2^ at 0.436 eV. For Sample B, it reduces from 2.82 × 10^13^ eV^−1^ cm^−2^ at 0.358 eV to 1.07 × 10^13^ eV^−1^ cm^−2^ at 0.436 eV. And for Sample C, it drops from 2.34 × 10^13^ eV^−1^ cm^−2^ at 0.358 eV to 1.04 × 10^13^ eV^−1^ cm^−2^ at 0.436 eV. Through comparison, it is found that the interface state densities of Sample A, Sample B, and Sample C decrease successively. This phenomenon indicates that increasing the ratio of DCS/NH_3_ during the LPCVD SiN_x_ growth process can effectively reduce the interface state density at the interface between the dielectric layer and the III-nitride. Specifically, during the formation of SiN_x_ through the reaction between DCS and NH_3_, Si predominantly forms Si–N and Si–H bonds with N and H. Increasing the proportion of DCS implies a relative decrease in the content of NH_3_ in the reaction process. It should be noted that the H that forms Si–H bonds with Si mainly stems from NH_3_ [29]. A reduction in the relative content of NH_3_ means that a significant amount of Si cannot participate in bonding. These unbonded or “free” Si atoms, acting as donors, penetrate into the interface between the dielectric layer and the III-nitride. Once in the interface, they fill a portion of the defects present, thereby reducing the interface state density [21].

## 4. Conclusions

In summary, this study investigated the effects of different growth conditions on the quality of LPCVD SiN_x_ dielectric layers, 2DEG characteristics, interface trap density, and devices’ performance when using LPCVD SiN_x_ as gate dielectric and surface passivation layers. By examining the impact of LPCVD SiN_x_ growth parameters on the growth rate and 2DEG properties, the growth conditions of LPCVD SiN_x_ were optimized. During LPCVD SiN_x_ growth, the surface potential of AlGaN barrier layers can be adjusted by regulating the gas flow ratio. Both Si-rich and N-rich SiN_x_ can reduce the surface potential and thereby enhance the 2DEG density. The pulse-mode I_DS_-V_GS_ method and frequency-dependent C-V measurement were employed to investigate the influence of different DCS/NH_3_ gas flow ratios on the interface trap density at the dielectric/III-nitride interface. In pulse-mode I_DS_-V_GS_ measurements, Sample C exhibited a Dit of 1.44 × 10^1^^2^ eV^−^^1^·cm^−^^2^ at reverse-sweep V_GS_base_ = 3 V, compared to 1.76 × 10^1^^2^ eV^−^^1^·cm^−^^2^ for Sample A. Frequency-dependent C-V measurements at a defect energy level of 0.436 eV showed interface state densities of 1.04 × 10^1^^3^ eV^−^^1^·cm^−^^2^ for Sample C and 1.55 × 10^1^^3^ eV^−^^1^·cm^−^^2^ for Sample A. Both characterization methods confirmed that higher DCS proportions effectively suppressed trap formation. The gas flow ratio during LPCVD SiN_x_ growth is a critical factor influencing dielectric layer quality. High-DCS-ratio SiN_x_ can effectively reduce interface trap density, but the increased gate leakage current caused by excessive Si donors must not be overlooked.

## Figures and Tables

**Table 1:** Gas flow ratio, temperature, pressure, refractive index, thickness, 2DEG density, mobility, and sheet resistance of AlGaN/GaN HEMT wafer with LPCVD SiN_x_.

	DCS/NH₃	T (°C)	P (mtorr)	Refractive Index	Thickness (nm)	N_s_ (×10^13^ cm^−2^)	µ_n_ (cm^2^/V∙s)	R_s_ (Ω/□)
Sample A	35/280	795	300	1.96	21	0.82 ± 0.03	1822 ± 20	496 ± 15
Sample B	70/280	795	300	1.99	22.5	0.86 ± 0.02	1882 ± 18	442 ± 12
Sample C	150/150	795	300	2.04	23	0.90 ± 0.02	1963 ± 26	388 ± 8
Sample D	150/75	795	300	2.05	22	0.91 ± 0.02	1992 ± 21	373 ± 14
Sample E	6/280	795	300	1.95	20	0.84 ± 0.02	1792 ± 22	462 ± 12
Sample F	70/280	780	300	1.99	23	0.85 ± 0.02	1852 ± 16	455 ± 10
Sample G	70/280	795	160	1.97	22	0.86 ± 0.02	1866 ± 23	448 ± 11
REF						0.73 ± 0.03	1690 ± 20	522 ± 9

**Table 2:** 2DEG density, mobility, and sheet resistance of AlGaN/GaN HEMT wafers with and without PECVD SiN_x_.

		N_s_ (×10^13^ cm^−2^)	µ_n_ (cm^2^/V∙s)	R_s_ (Ω/□)
Sample H	Without SiN_x_	0.73 ± 0.02	1902 ± 21	443 ± 12
	With SiN_x_	0.88 ± 0.02	1744 ± 23	418 ± 11
Sample I	Without SiN_x_	0.83 ± 0.03	1837 ± 22	407 ± 11
	With SiN_x_	0.88 ± 0.03	1778 ± 26	372 ± 10
Sample J	Without SiN_x_	0.81 ± 0.02	1670 ± 23	488 ± 13
	With SiN_x_	0.87 ± 0.02	1590 ± 21	453 ± 12

## Data Availability

The original contributions presented in this study are included in the article. Further inquiries can be directed to the corresponding author.
